# Behavioral response of naïve and non-naïve deer to wolf urine

**DOI:** 10.1371/journal.pone.0223248

**Published:** 2019-11-27

**Authors:** Hermine Annette Lisa van Ginkel, Christian Smit, Dries Pieter Jan Kuijper

**Affiliations:** 1 Conservation Ecology Group, Groningen Institute for Evolutionary Life Sciences, University of Groningen, Groningen, The Netherlands; 2 Mammal Research Institute, Polish Academy of Sciences, Białowieża, Poland; Michigan Technological University, UNITED STATES

## Abstract

Large carnivores are recolonizing many regions in Europe, where their ungulate prey have lived without them for >150 years. Whether the returning large carnivores will modify ungulate behavior and indirectly affect lower trophic levels, depends on the ability of ungulates to recognize risk based on past encounters and cues indicating carnivore presence. In two case studies, we tested, by means of camera trapping, the behavioral response of deer to wolf urine. The first case study was in the Netherlands where deer (still) live in absence of wolves, and the second in Poland with long-term wolf presence. As controls we used water (no scent) and all-purpose soap (unfamiliar scent). Deer vigilance level on control plots was 20% in both case studies indicating that wolf occupancy *per se* does not lead to a consistent difference in behavior. Placing wolf urine did not significantly affect deer behavior in either the wolf-absent or the wolf-present area. More intense cues, or a combination of cues, are likely needed to affect deer behavior. Moreover, we found an unexpected reaction of deer towards all-purpose soap of reduced foraging (and tendency for increased vigilance) in the wolf-present area, whereas it did not affect deer behavior in the wolf-absent area. We hypothesize that deer associate all-purpose soap with human presence, causing no response in human-dominated landscapes (the Netherlands), but triggering a behavioral reaction in more remote areas (Poland). This illustrates attention should be paid to controls used in scent experiments as they may be associated differently than intended.

## Introduction

As a result of land abandonment and improved protective legislation [1–3], large carnivores are recolonizing many regions in Europe after approximately 150 years of absence [4]. The impact of these returning large carnivores on ecosystem functioning depends in part on the reaction of ungulates to the re-establishment of the landscape of (perceived) predation risk. To reduce predation risk, ungulates can adjust their spatial and temporal distribution [5–7], increase vigilance and reduce foraging effort [8,9], and increase group size in high risk areas [10]. These behavioral changes can affect their physiology and demography in the long term [11]. Moreover, these changes in ungulate behavior can lead to spatial variation in foraging intensity, resulting in changes in vegetation growth and composition at the landscape level [12–14]. Hence, it is important to know how ungulates that have lived for generations in absence of predators react to large carnivores upon their return.

According to the optimal foraging theory, ungulates should select for foraging patches with the highest energy return [15]. In presence of large carnivores ungulates face a trade-off between food acquisition and risk avoidance [16,17]. Both wild and domesticated ungulates coexisting with large carnivores generally show increased vigilance in high predation risk areas [18–21] leading to reduced food intake. Moreover, spatial avoidance of high risk areas leads to increased energy expenditure and sub-optimal resource use [22–24]. Therefore, antipredator behavior is costly and should be selected against in a predator-free environment. As a consequence, ungulates are predicted to become naïve or less sensitive to predator cues after several generations in the absence of large carnivores [25–28]. However, there can be a costly time lag before naïve ungulates learn how to respond properly to carnivores once they return to a predator-free area [26,29–31]. Moreover, prey species are predicted to lose their anti-predator behavior towards a historical predator only when there are no other predators of the same archetype present in the system [32,33]. These factors can explain why prey species seem to have become naïve in some cases, while maintaining their anti-predator behavior even in the absence of their predator in other cases.

Indeed, several studies found no behavioral response of ungulates towards large carnivore cues in predator-free areas and therefore concluded that these ungulates had become naïve [25,26,34]. For example, moose (*Alces alces*) living in areas without large carnivores for 40 to 75 years showed little response to olfactory and acoustic gray/grey wolf (*Canis lupus*) cues [26]. In contrast, several other studies showed that wild and domesticated ungulates do not always lose their antipredator behavior and are able to recognize predator cues and react to them, despite a long absence of large carnivores [35–41]. For example, after being isolated for 1200 years from their ancestral predators, Père David’s deer (*Elaphurus davidianus*) still responded strongly to the tiger (*Panthera tigris*), with increased vigilance to and avoidance of visual and acoustic cues [38]. These contrasting results make it difficult to predict whether ungulates in large carnivore-free areas are naïve and how sensitive they will be when large carnivores recolonize these areas [32,42].

The objective of this study was to investigate, in two separate case studies, the behavioral response of deer to wolf urine, as an olfactory predator cue. In the first case study we tested how wolf urine affects the behavior of deer living in National Park Veluwezoom, the Netherlands. In the Netherlands wolves have been absent for 150 years, but are currently on the fringe of recolonization. Therefore we wanted to test how the deer react to an olfactory wolf cue indicative for their naivety. In the second case study we tested how wolf urine affects the behavior of deer in the Białowieża forest, Poland, where wolves and deer have co-occurred for more than 100 years [43]. We expected that deer in the wolf-absent area have lost their costly antipredator behavior, have become naïve, are less vigilant and therefore will not respond to wolf urine. By contrast, we expected the deer in the wolf-present area have a higher background vigilance level and show a strong increase in vigilance as response to wolf urine, since they associate this scent based on experience with potential risk.

## Materials and methods

### Ethics statement

Permissions to carry out this experiment in National Park Veluwezoom, the Netherlands, was granted by the management of the National Park. To conduct this experiment in the managed part of the Białowieża forest the Polish State Forestry (Białowieża district) granted permission. Since all data collected on vertebrates (ungulates) was based on non-invasive sampling by camera trapping, no permission from ethical commissions was required. The used wolf urine was purchased via a company that collects urine from animals in game farms, zoos and preserves. State agencies conduct regular inspections of these facilities to assure that the facility meets all health and treatment standards. The wolf urine is collected in a non-invasive way via a floor drainage system in the animal cages.

### Study area

We performed a scent experiment in the spring of 2016 in two different areas that we treat as two separate case studies. Case study 1 is performed in National Park Veluwezoom (NPV), the Netherlands. In the Netherlands, the last wolf of was shot in 1869 (www.wolveninnederland.nl) and therefore wolves are believed to have been absent for almost 150 years. However, wolves are recolonizing Europe and are on the fringe of the Netherlands. The first modern sighting of a grey wolf in the Netherlands was in 2015. Case study 2 was performed in the Białowieża Primeval forest (BPF), Poland, where deer and wolves have co-occurred for more than 100 years [43]. During the last two centuries, there have been short periods (1882–1915 and 1958–1970 see Jedrzejewska et al. [43]) when wolf densities in the BPF were very low, but we assume these periods are too short to influence the antipredator behavior of the ungulates.

The Dutch NPV (52°04’N, 6°01’E) is a national park of 51 km^2^, consisting of forest (ca. 34 km^2^) and heathland. The NPV is grazed by Highland cattle (*Bos taurus*), Icelandic horses (*Equus ferus caballus*), red deer (*Cervus elaphus*, 7.1 individuals km^-1^), fallow deer (*Dama dama*, 5.1 ind. km^-1^), roe deer (*Capreolus capreolus*) and wild boar (*Sus scrofa*, 7.1 ind. km^-1^, unpublished data). The only meso-carnivores present are red fox (*Vulpes vulpes*) and martens (*Martes* sp.). Within the NPV seasonal hunts take place to control the deer populations in autumn and winter. The Polish BPF (52°45’N, 23°50’E), is 600 km^2^ and consists of a National Park (102 km^2^) and an adjacent managed forest. This experiment was conducted in the managed part of the BPF that is owned by the State Forestry and therefore human activities (logging and seasonal hunting) are allowed. The BPF has a varied native ungulate assemblage consisting of red deer (4.7 ind. km^-1^), wild boar (3.2 ind. km^-1^), roe deer (0.8 ind. km^-1^), bison (*Bison bonasus*, 0.8 ind. km^-1^) and moose (0.06 ind. km^-1^, [44]). These ungulates co-occur with their natural predators wolf (2–3 ind. per 100 km) and lynx (*Lynx lynx*, 1–3 ind. per 100 km, [45]) and the meso-carnivores red fox, martens and raccoon dog (*Nyctereutes procyonoides*). In the BPF wolf predation is the most important mortality factor for red deer and the second most important factor for roe deer mortality [46,47].

The deer species that are present differs between the BPF and the NPV. Red deer occurs in both areas and is the main prey for wolves in the BPF. Roe deer, the second prey for wolves in the BPF, also occurs in both areas however we recorded too few visits to allow for a statistical analysis. Fallow deer occurs only in the NPV and is introduced as hunting game in the 15^th^ century. Therefore we decided to conduct the statistical analysis on red deer behavior only, thereby excluding roe deer and fallow deer.

### Experimental set-up and camera placement

**Fig 1:**
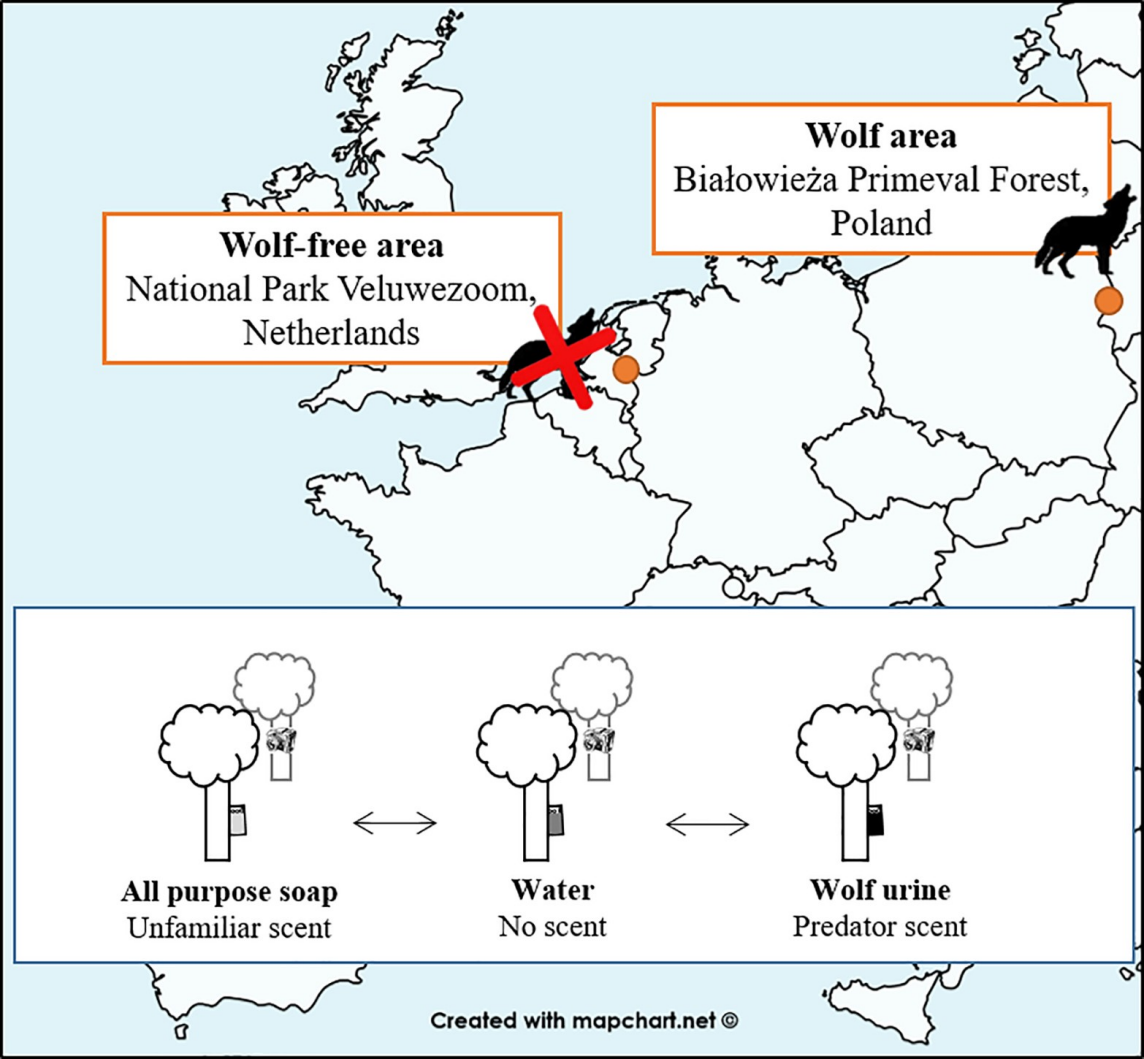
Design of our olfactory cue experiment in both case studies. In the wolf-absent National Park Veluwezoom (NPV), The Netherlands, deer live in absence of wolves for 150 years. In our wolf-present area the Białowieża Primeval forest (BPF), Poland, deer live along wolves for more than 100 years. In both study areas we established per location three scent plots at least 50 m apart to avoid one treatment influencing the other. The scent order was randomly decided. On each plot we placed a dispenser (small green plastic tube of 30 ml with two holes at the top) containing the scent and a camera trap at a tree directed towards the dispenser to monitor red deer behavior. We replicated this 25 times in the NPV and 20 times in the BPF.

We selected locations a minimum of 50 m from forest roads and fences, and with clear signs of recent deer activity (fresh droppings, tracks or browsing marks) to increase the chance of capturing deer on camera. The distance between locations was a minimum of 200 m. Per location three plots were established, at least 50 m apart to avoid one treatment influencing the other (in line with Kuijper et al. [18]; Fig 1). In the study of Kuijper et al. [18] the distance between the paired plots (wolf scat versus control) was 50–500 meters and resulted in a clear difference in red deer behavior. A similar set-up was used in Wikenros et al. [19] and a clear difference in behavioral response of both red and roe deer towards lynx scats and control plots was observed. This implies that a distance of 50 meters between paired plots is large enough to prevent airborne scent contamination. Moreover, the results of Kuijper et al. [18] and Wikenros et al. [19] suggest a very fine-scale risk effect of circa two meters from the wolf scat on increased perceived predation risk. On each plot a dispenser was taped to a tree at a height of 1–1.2 meter. Dispensers consisted of a small green plastic tube of 30 ml with two holes at the top, for a constant and sufficient evaporation of scent and a lid as protection from rain. A camera trap (Ecotone in the NPV and Bushnell Trophy Cam HD in BPF) was mounted at a height of 1 meter at a tree directed towards the dispenser at a distance of 8–10 meters. Within each study area the same camera trap type was used, and since the NPV and BPF are two separate case studies they were not directly compared in the statistical analysis. Kuijper et al. [18] and Wikenros et al. [19], showed that deer have the strongest response to wolf scent in the first week after which the behavioral response attenuates. Therefore we monitored our plots for seven consecutive days, and new scent plots were set-up at new locations subsequently.

At each location we used three different olfactory cues in the dispensers. As predator scent pure wolf urine (ordered via www.roofdierpis.nl, a Dutch subsidiary of www.predatorpee.com; Maine Outdoor Solutions, 2706 Union St., Hermon, Maine 05501 USA) was used. As control scents we placed water and all-purpose soap (with lemon scent). Water was used to record deer behavior in absence of a predator scent and to control for possible behavioral responses to our scent or presence after placing the dispensers. All-purpose soap was used to determine the response of red deer to an unfamiliar scent, to compare this with the unfamiliar wolf urine scent for red deer living in the wolf-absent area. It was randomly decided which olfactory cue was placed at which plot within each location. After seven days the dispensers still contained 15 ml of the scents and this did not differ between treatments or the two case studies (personal observations), indicating comparable evaporation between locations and sites. The olfactory cue was thus present for the whole experimental period, assuming no temporal effects in scent intensity.

#### Case study 1: Deer behavior in response to wolf urine in wolf-absent area

In NPV (April 22 –July 8, 2016) the dispensers and camera traps were placed along tracks used by deer based on fresh droppings and tracks, mainly on the edge of forest to open areas. The canopy was dominated by Scottish pine (*Pinus sylvestris*), silver birch (*Betula pendula*) and pedunculate oak (*Quercus robur*), whereas the herbaceous layer was dominated by bilberry (*Vaccinium myrtillus*) and grasses. The canopy openness of the plots was 35.7 ± 1.5% (mean ± SE) and visibility was 30.7 ± 1.1 m (Table A in S1 File). In the NPV the experiment was replicated 25 times leading to 75 experimental plots in total. Eighteen locations were within 200 m from each other due to limited space, but these were used with a six week interval so we treat these as independent measurements.

#### Case study 2: Deer behavior in response to wolf urine in wolf-present area

In the BPF (May 5 –June 24, 2016) the dispensers and camera traps were placed in natural forest gaps with intensively-browsed hornbeam (*Carpinus betulus*) saplings in the *Tilio-Carpinetum* forest. Since the BPF is a rather closed forest system with only small clearings, forest gaps are preferred for foraging by deer [48]. The canopy of our selected locations was dominated by Norway spruce (*Picea abies*), pedunculate oak, hornbeam and Scottish pine, whereas hornbeam saplings (< 2 m), grasses, ferns and raspberry (*Rubus idaeus*) dominated the ground layer. The canopy openness of used plots was 49.1 ± 2.4% and the visibility 21.3 ± 1.4 m (Table A in S1 File). The experiment was replicated 20 times in the BPF leading to 60 experimental plots in total. Wolves are present in the entire BPF, however they are most active in the core of their territory. For that reason, the plots of our experiment were located outside the (risky) core which in general reduced the perceived risk for deer. Moreover, the camera traps recorded no wolf or lynx on our plots, which means we did not attract large carnivores that could enhance perceived risk on wolf urine plots [49].

### Behavioral classification

All recorded videos were analyzed for species and number of individuals per visit, visitation frequency, visitation length and behavior of the red deer visiting our plots. Consecutive videos recorded within a five minute interval were grouped and analyzed as one visit. Red deer behavior was scored based on the classification used by Kuijper et al. [18] with one additional category (nr. 3): 1) *foraging*—deer is grazing (eating grass or herbs) or browsing (woody species) with its head below shoulders, 2) *vigilant*—individual is standing still with its head above shoulders, looking around and/or twitching ears, without chewing, 3) *vigilant while foraging*: deer is chewing while standing still with its head above shoulders, 4) *walking*—while not foraging or chewing, 5) *running*—while not foraging or chewing, 6) *sniffing*—defined as animal moving head up and down with stretched neck, without chewing, when smelling, 7) *sudden rush*—when an animal went from standing still to running (abrupt response), 8) *vigilant towards camera*—walking towards camera and sniffing it or during dark hours were vigilant towards infrared light of camera, 9) *other*—included all other types of behavior such as scratching, licking. We determined the time (in seconds) an individual was showing a certain behavior and calculated the proportion of time for each type of behavior of the total time behavior was visible. Visitation length (total time in seconds on plot) for each individual was determined including the time behavior was not visible (e.g. when individual was temporally hidden behind a tree or shrub, or on night-time recordings). Visitation frequency was calculated as the number of red deer visits per day (total number of visits per plot divided by the number of actual recording days—usually seven days, occasionally one or eight).

### Statistical analysis

We could not statistically compare the red deer behavioral responses towards the scent treatments between the two case studies with a 2-way ANOVA as this would violate the assumption of normal distributed residuals. We explored the possibility for a non-parametric equivalent with the ARTool-package [50], however we could not solve the problem indicated by a warning message which makes the outcomes unreliable. Due to these statistical constraints and the earlier mentioned habitat differences between the two case studies, we argue it is more appropriate to use the non-parametric Kruskal-Wallis test. For both case studies we tested for differences between the treatments in visitation frequency (nr. of visits day^-1^), visitation length (nr. of seconds on plot) and red deer behavior (proportion of time spend vigilant, foraging, walking, running or sniffing).

Often the camera recorded a group of red deer. To avoid pseudoreplication, the behavior of only one individual per group visit was included to analyze the treatment effect on visitation length and deer behavior. Whether we analyzed the first individual appearing on camera or the individual longest present on the plot did not change the results. Therefore we will present the results of the individual that was longest present on the plot only.

For the behavioral analysis we included only videos when an individual was present for > 4 seconds, to exclude videos in which red deer were only running or walking by. The behavior *vigilant while foraging* was rarely scored and therefore grouped with *foraging*. We choose to combine *vigilant while foraging* with *foraging* and not with *vigilant* as being vigilant without chewing (i.e induced vigilance) is more costly as it results in lost foraging opportunities compared to vigilant while chewing (i.e. routine vigilance, following Blanchard and Fritz [51]). For the same reason *vigilance towards camera* was grouped with *vigilance*. Due to low and unequal numbers of recording we could not test for differences in behavior between sexes (Table B in S1 File). The calculated average time spent on a particular behavior is therefore a mix of both sexes. Calves were excluded from the analysis, as their behavior is assumed to be mainly determined by their mother [18]. The statistical analyses were done in RStudio with R-3.6.0 (R Core Team 2019).

## Results

### Case study 1: Red deer behavior in response to wolf urine in wolf-absent area

In the wolf-absent area we recorded in total 69 red deer visits during a total of 507 trapping days. For more information about the sex-distribution of the red deer see Table C in S1 File.

**Fig 2:**
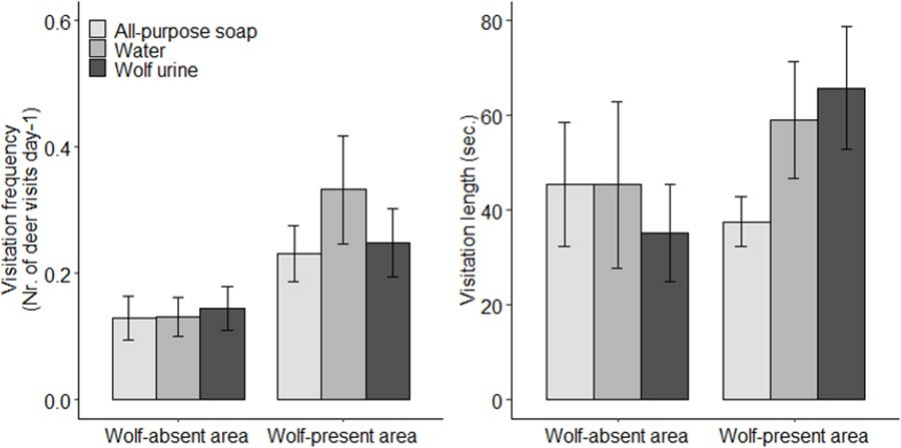
Visitation frequency and visitation length (mean ± SE) on the treatment plots for both study sites. Wolf-absent case study: treatment has no significant effect on visitation frequency (*H*(2) = 0.096, *P* = 0.953) nor visitation length (*H*(2) = 0.840, *P* = 0.657). Wolf-present case study: treatment has no significant effect on visitation frequency (*H*(2) = 0.153, *P* = 0.926) nor visitation length (*H*(2) = 2.390, *P* = 0.303).

Red deer visited a scent plot on average once every six days (average across treatments: 0.16 ± 0.02 SE), with no difference in visitation frequency between treatments (*H*(2) = 0.096, *P* = 0.953; Fig 2), or in visitation length (41.7 ± 8 seconds (mean ± SE); *H* (2) = 0.840, *P* = 0.657; Fig 2).

**Fig 3:**
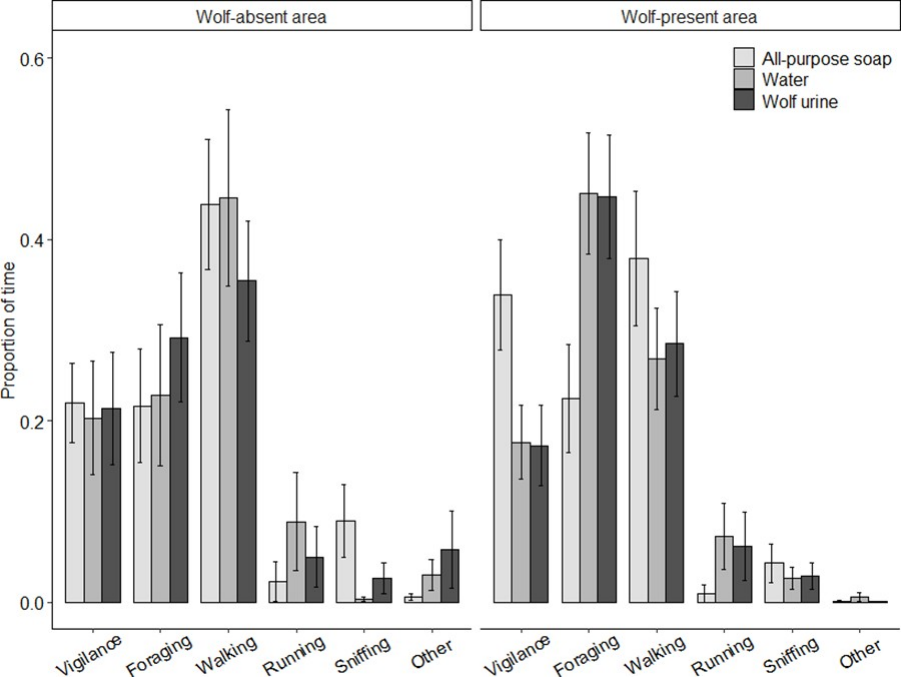
Behavioral response of deer to all-purpose soap, water and wolf urine in both study sites. Average proportion of time red deer in wolf-absent case study (left panel) and the wolf-present case study (right panel) spent on different behavior categories. None of the treatments significantly affected the behavior of red deer living in the wolf-absent area. In the wolf-present area the proportion of time red deer spend on foraging was significantly lower on all-purpose soap plots (*H*(2) = 7.088, *P* = 0.029), whereas the other behavior categories were not affected by our treatments.

On the water plot in the wolf-absent area red deer spent approximately 20% of their time on vigilance, 23% on foraging, <1% on sniffing, 45% on walking and 9% on running. Sudden rush was scored 11 times (wolf urine plot: 5 times, soap plot: 4, and water plot: 2). However, none of the behavioral categories was significantly affected by our treatment (*vigilance*: *H*^2^(2) = 0.695, *P* = 0.707; *foraging*: *H*(2) = 1.001, *P* = 0.606; *sniffing*: *H*(2) = 3.284, P = 0.194: *walking*: *H*(2) = 0.899, P = 0.638; *running*: *H*(2) = 0.606, P = 0.739; Fig 3). In general we could observe clear reactions to treatments, but there was a large variation in the response (see Video Compilation in S2 File and Table D in S1 File).

### Case study 2: Deer behavior in response to wolf urine in wolf-present area

In the wolf-present area we recorded in 415 trapping days a total of 115 red deer visits (for more and detailed information see Table B and Table C in S1 File).

Visitation frequency was approximately one visit every four days (average across treatments 0.27 ± 0.04 SE), and did not differ between treatments (*H* (2) = 0.153, *P* = 0.926; Fig 2). Visitation length was on average 55.1 seconds (± 6.6), with the lowest average visitation length on the all-purpose soap plot (37.6 ± 5.2), but this was not significantly different between the treatments (*H* (2) = 2.390, *P* = 0.303; Fig 2). On the water plot red deer were approximately 18% of the time vigilant, 45% of the time was spent foraging, 3% sniffing, 27% walking and 6% running. We found a trend of ca. 16% more vigilance on all-purpose soap plots (33.9 ± 6 SE), compared to the wolf urine or the water plots (17 ± 4 SE and 18 ± 4, respectively, *H*(2) = 4.861, *P* = 0.088; Fig 3), which resulted in a reduction in foraging of approximately 23% on all-purpose soap plots (22 ± 6 SE) compared to wolf urine (45 ± 7 SE) and water (45 ± 7 SE) plots (*H*(2) = 7.088, *P* = 0.029). Proportion of time spend on sniffing, walking or running was not affected by our treatments (*H*(2) = 0.639, *P* = 0.727; *H*(2) = 1.769, *P* = 0.413, and *H*(2) = 2.511, *P* = 0.285 respectively). Sudden rush was scored 8 times in total (wolf plot: 4 times, soap plot: 2 and water plot: 2).

## Discussion

The return of large carnivores to areas where they have been absent for a long time, raises the question whether prey species will recognize and respond adequately to them once they return. Studies on prey naiveté in response to predator cues show mixed outcomes [26,31,52, 53]. Whether prey will recognize their historical or a non-native predator and respond effectively towards it, mainly depends on the eco-evolutionary experience of the prey with predators [32,42,54]. In case study 1, we tested whether deer in the Netherlands still show a behavioral response towards the scent of wolves, their historic predator, and in case study 2 we performed the same experiment in Poland were deer and wolf have long coexisted. Our study showed that: 1) wolf urine did not affect the behavior of red deer either living in a wolf-absent or wolf-present area; 2) the average vigilance level of red deer was 20% irrespective of wolf occurrence, and 3) there is a trend that all-purpose soap increased vigilance only of red deer in the wolf-present area (Poland, case study 2), which came at the costs of a reduction in foraging time.

### No behavioral response of red deer to wolf urine

Wolf urine did not affect the red deer behavior in the wolf-absent area. Our result corresponds to Elmeros et al. [34], who also found no effect of wolf urine on red deer or roe deer behavior in an area where wolves are absent. Several studies have suggested that ungulates can lose their antipredator behavior after living in absence of their natural predator for multiple generations [25,26,42]. Our results from case study 1, could indicate that red deer in the wolf-absent area did not recognize the scent of their ancestral predator and therefore are naïve (level 1 naiveté; [55]). However, it would be too simple to directly conclude that red deer in the wolf-absent area are naïve only because we did not observe an increase in vigilance behavior.

An alternative hypothesis for the lack of behavioral response in the wolf-absent area is that the red deer associate the wolf urine with domestic dog (*Canis lupus familiaris*) presence. The urine of different carnivore species contains similar chemical components that species can respond to [56]. Wolves and dogs are closely related and therefore red deer may react in a similar way to urine or other scent cues from wolves and dogs (see Arnould et al. [57]). Carthey et al. [58] showed that the closely related dog and dingo (*Canis lupus dingo*) also have similar scent profiles, which make recognition of the main predator difficult for their prey species [59]. Since leashed dogs (stray dogs do not occur in the NVP) are fairly common in our wolf-absent area, red deer are supposedly well-acquainted to canine scent and therefore might not respond to the wolf urine (level 2 naiveté; [55]), as red deer might have learned that the only canines present (leashed dogs) impose no threat [60]. This can imply that the chemical profiles of the closely related wolf and dog are so similar that red deer might not be able to distinguish the urine of the ‘dangerous’ wolf from the ‘harmless’ dog.

In contrast to our prediction, also the red deer from case study 2 living in a wolf-present area did not adjust their behavior on wolf urine plots. This is surprising as in the BPF the red deer is the main prey for wolves [61], and wolves are the main natural mortality factor for deer [44]. Hence the relevance for red deer to react to cues indicating potential predation risk by wolves in the BPF. Our reasoning for the lack of behavioral response to wolf urine in the wolf-absent case study cannot be applied to our wolf-present case study. Therefore, we need to explore alternative hypotheses that can explain why red deer did not adjust their behavior in response to wolf urine, in both the wolf-absent and the wolf-present case study.

We used 30 ml of wolf urine and found no significant behavioral response in deer. Parsons and Blumstein [62] found reduced foraging and increased number of flights in kangaroo (*Macropus* sp.) using 40 ml of dingo (*Canis lupus dingo*) urine, and 7 ml of European lynx (*Lynx lynx*) urine was already enough to increase roe deer vigilance [63]. Also, Rouco et al. [64] observed spatial avoidance by European rabbits (*Oryctolagus cuniculus*) after 4 ml of red fox (*Vulpes vulpes*) scent was placed on a plot. Under natural conditions, wolf scent marking via urination is estimated at 5 ml urine [65]. The risk cue we applied did result sometimes in a behavioral response, as we did score *sudden rush* (an abrupt response from standing still to running) slightly more on wolf urine plots in comparison to all-purpose soap and water plots in both case studies (but due to low sample size this was not statistically tested). To give an impression we made a compilation of 17 recorded videos (attached as Video in S2 File, and see Table D in S1 File for description) that shows a wide variety of behavioral responses. These observations suggest wolf urine can elicit a fear response (both in Polish and Dutch deer), however only when deer were within 0.5 m of the dispensers. This explains its efficacy as repellent to reduce browsing damage when applied on or in direct vicinity of a foraging place [35,66,67]. We argue that the scent intensity applied to our plots was sufficient to be noticed by the deer, but since the scent was offered in a dispenser, it may not have been strong or widespread enough to induce a behavioral response in the full detection range of the camera.

Previous studies showed that deer in the Białowieża forest are responsive to olfactory cues and increased their vigilance levels by 2-fold in response to wolf scats [18] and reduced visitation length in response to lynx scats [19]. This suggests that a large carnivore scat creates a stronger predation risk cue than the scent of urine. Scats might provide longer scent in the environment and provide additionally a visual cue. Also in other studies, ungulate risk-related behavior was observed more often when both scats and urine were used [20,37,39] or were combined with visual or audible cues [36,38,40,68]. Deer likely use a whole array of cues (olfactory, visual and audible) and landscape features to estimate risk [53,54,69]. The accumulation of olfactory (urine, scat, territorial scratching, scent glands), visual (wolf sightings), and auditory cues (wolf howling) creates a spatial pattern indicating wolf space use (see for example [70]). Ungulate prey species may use this information to determine high and low risk areas. Thus, it is likely that a more complete set of cues–rather than one very local olfactory cue–in combination with spatially varying levels of intensity, is needed to affect prey behavior [71].

Finally, behavioral responses can depend on the freshness of the cue [18,19,72]. Fresh urine hints that the predator might still be in vicinity, indicating high potential predation risk. Urine freshness rather than quantity might therefore be a more relevant factor prey species are principally reacting to [72]. The wolf urine used in our study was some months old, but to what extent this may have affected our results is unclear (we did observe some clear responses to this wolf urine, see Video Compilation in S2 File).

### Deer vigilance level in the wolf-absent and wolf-present area

Irrespective of the area (wolf-absent or present), the average vigilance level of red deer was approximately 20%, which is equivalent to vigilance levels of deer in comparable scent experiments [18,39,60]. This raises the question of what mechanism determines vigilance levels. A possible answer is that disturbance frequency in general is an important driver for vigilance. It has been shown that the vigilance level of elk depends on the frequency of presence or disturbance of wolves in an area [6]. In our wolf-absent study area, the lack of wolf presence could have been replaced by more human presence, explaining comparable vigilance levels in both case studies. Deer can increase their vigilance levels or adjust their temporal and spatial activity patterns in response to roads and human activities like hunting, hiking and logging [73–79]. Recent studies showed that the influence of humans on ungulate distribution and behavior can overrule the effects of natural predators [77–82]. Apparently, human disturbance can have a large influence on deer behavior and probably is driving deer vigilance levels in large carnivore-free areas.

The 20% levels of vigilance might additionally explain why red deer did not respond to wolf urine: the wolf urine is not a large enough trigger to increase the already present level of vigilance. A further increase in vigilance might be too costly, because it would result in a larger reduction in foraging time [9].

### Deer response in anthropogenic versus more remote landscapes

The unambiguous results of our study indicate that it is important to carefully consider the treatments to control for the effect of introducing an unfamiliar/novel scent while doing scent experiments. Prey species may simply show elevated vigilance levels (interpreted as a fear response) because they encounter an unfamiliar scent. To control for this we used all-purpose soap as unfamiliar but not risky scent. The Dutch red deer (wolf-absent area) did not adjust their behavior on the all-purpose soap plots, but we observed a change in behavior by Polish red deer (wolf-present) with reduced foraging and a trend towards increased vigilance on the soap plots. This pattern in behavior towards all-purpose soap is probably explained by the difference in human population densities between our two case studies.

Dutch deer could be more used to human disturbance due to the high number of tourists (2 million inside the NPV) that yearly visit this relatively small national park (pers. comm. with the managers) compared to the estimated 200.000 tourists in BPF concentrated around the Białowieża village (pers. comm. K. Niedzialkowski and W. Walankiewicz). Due to the higher number of visitors and consequently the exposure to human disturbance, Dutch deer are more used to the array of human-derived scents than Polish deer. As a result, Dutch red deer might not perceive all-purpose soap as unfamiliar scent, but associate it with human presence, whereas all-purpose soap is more unfamiliar for Polish red deer due to the general low exposure of Polish red deer to anthropogenic scents. A recent study showed indeed that in anthropogenic areas compounds of silicone oils used in cosmetic and cleaning products are an important player in urban emission [83]. Several studies showed that mammals living in anthropogenic landscapes can get used to human presence and adapt accordingly, showing different behavior than their rural counterparts [84,85]. The influence of human activity on mammal behavior should not be underestimated when performing research in anthropogenic landscapes. In other olfactory cue studies gasoline or eau de cologne [39] were used as unfamiliar, not-risky scents, though these scents can likewise be associated with human presence. We argue that all-purpose soap, gasoline and eau de cologne may function well in remote areas as unfamiliar scents, but may function less in human-dominated areas where they may be associated with human presence.

Carthey and Banks [54] already stated we should be careful with interpreting the outcomes from scent-experiments, and our study also shows that the results might not be so straightforward. In many regions, human activities play a pronounced role by influencing both large carnivore and ungulate behavior and distribution [25,77,79,8,86–88]. The influence of humans, and their scent, should not be underestimated in olfactory cue experiments, and should thus be incorporated in future studies.

## Supporting information

S1 File **Table A. Canopy openness (%) and visibility (m) on the scent plots in wolf-absent site (National Park Veluwezoom, NPV, the Netherlands, case study 1) and the wolf-present site (Białowieża Primeval Forest, BPF, Poland, case study 2)**.
**Table B. Number red deer male and female visits recorded in the wolf-absent site, the Netherlands, and in the wolf-present site, Poland**.
**Table C. Number of deer visits for both case studies split per sex and deer species.** Taking only visits into account when deer was > 4 seconds on plot, since that subset was used for statistical analysis.
**Table D. Description of videos selected for the compilation to show the variety of observed behaviors towards the three scent treatments.** In the attached Video Compilation in S2 File we indicated the study site and the treatment (top left) along with a number (top right) that corresponds to the video number in this table. In video 5 a male red deer is foraging near the dispenser, lifts his head, sniffs and runs off. We scored this type of behavior as *sudden rush* (an abrupt response from standing still to running, see also video 16) and observed it slightly more on wolf urine plots in comparison to all-purpose soap and water plots in both case studies (but due to low sample size this was not statistically tested). Moreover we observed deer started walking backwards or walked around the tree after they sniffed all-purpose soap or wolf urine (video 12 and 14).
(DOCX)

S2 File Video Compilation of 17 recorded videos showing a wide variety of behavioral responses. See Table D in S1 File for a description.
(MPG)
