# Supplementary material for: Behavioral response of naïve and non-naïve deer to wolf urine
Source: PLoS One. 2019 Nov 27;14(11):e0223248. doi: 10.1371/journal.pone.0223248 (PMC6880981; doi:10.1371/journal.pone.0223248)
Supplement: S1 File — Table A. Canopy openness (%) and visibility (m) on the scent plots in wolf-absent site (National Park Veluwezoom, NPV, the Netherlands, case study 1) and the wolf-present site (Białowieża Primeval Forest, BPF, Poland, case study 2). Table B. Number red deer male and female visits recorded in the wolf-absent site, the Netherlands, and in the wolf-present site, Poland. Table C. Number of deer visits for both case studies split per sex and deer species. Taking only visits into account when deer was > 4 seconds on plot, since that subset was used for statistical analysis. Table D. Description of videos selected for the compilation to show the variety of observed behaviors towards the three scent treatments. In the attached Video Compilation in S2 File we indicated the study site and the treatment (top left) along with a number (top right) that corresponds to the video number in this table. In video 5 a male red deer is foraging near the dispenser, lifts his head, sniffs and runs off. We scored this type of behavior as sudden rush (an abrupt response from standing still to running, see also video 16) and observed it slightly more on wolf urine plots in comparison to all-purpose soap and water plots in both case studies (but due to low sample size this was not statistically tested). Moreover we observed deer started walking backwards or walked around the tree after they sniffed all-purpose soap or wolf urine (video 12 and 14). (DOCX) [file pone.0223248.s001.docx]

| **S1 Table A.** |
| --- |

|  |  | Canopy openness (%) | | Visibility (m) | |
| --- | --- | --- | --- | --- | --- |
|  |  | Mean ± SE | Range | Mean ± SE | Range |
| Wolf-absent  (NPV) | All-purpose soap | 37.4 ± 3.4 | 10 – 96 | 29.8 ± 1.8 | 16 – 48 |
|  | Water | 34.2 ± 2.0 | 10 – 58 | 31.0 ± 1.7 | 20 – 45 |
|  | Wolf | 35.6 ± 2.2 | 16 – 74 | 31.2 ± 2.3 | 6 – 50 |
|  | Treatments grouped | 35.7 ± 1.5 | 10 – 96 | 30.7 ± 1.1 | 6 – 50 |
|  |  |  |  |  |  |
| Wolf-present (BPF) | All-purpose soap | 41.9 ± 3.4 | 15 – 68 | 21.8 ±2.3 | 7 – 44.7 |
|  | Water | 50.5 ± 4.5 | 20 – 94 | 20.2 ± 2.5 | 6.4 – 59.8 |
|  | Wolf | 54.9 ± 4.4 | 13 – 94 | 21.7 ± 2.5 | 7.4 – 54.3 |
|  | Treatments grouped | 49.1 ± 2.4 | 13 – 95 | 21.3 ± 1.4 | 6.4 – 59.8 |

S1 Table B.

|  | Wolf-absent | Wolf-present | |
| --- | --- | --- | --- |
| Total number of trapping days | 507 | 415 | |
| Badger (*Meles meles*) | 7 | 2 | |
| Deer species unknown | 15 | 0 | |
| European bison (*Bison bonasus*) | – | 19 | |
| European hare (*Lepus europaeus*) | 5 | 0 | |
| Fallow deer (*Dama dama*) | 21 | – | |
| Highland cattle (*Bos taurus*) | 5 | – | |
| Icelandic horse (*Equus ferus caballus*) | 32 | – | |
| Moose (*Alces alces*) | – | 3 | |
| Pine marten (*Martes martes*) | 6 | 0 | |
| Red deer (*Cervus elaphus*) | 69 | 121 | |
| Red fox (*Vulpes vulpes*) | 6 | 4 | |
| Roe deer (*Capreolus capreolus*) | 39 | 5 | |
| Wild boar (*Sus scrofa*) | 99 | 10 | |
| - Means the species does not occur in the area | | |  |

| **S1 Table C** | | | |
| --- | --- | --- | --- |
|  | All-purpose soap | Water | Wolf urine |
| **Wolf-absent** | **18** | **18** | **19** |
| *Female* | *15* | *13* | *13* |
| *Male* | *3* | *5* | *6* |
| **Wolf-present** | **29** | **35** | **30** |
| *Female* | *17* | *18* | *16* |
| *Male* | *12* | *17* | *14* |

| **S1 Table D** | | | |
| --- | --- | --- | --- |
| Study site | Treatment | Video Nr. | Observed behavior |
| Białowieża forest, Poland –  Wolves present | Water | 1 | Group of female red deer with calf walking by 🡪 no response |
|  |  | 2 | Male red deer walking 🡪 no response |
|  | All-purpose soap | 3 | Male red deer sniffing 🡪 avoiding bush with scent |
|  |  | 4 | Female red deer walking 🡪 no response |
|  | Wolf urine | 5 | Male red deer foraging near scent, head goes up, sniffs 🡪 sudden rush |
|  |  | 6 | Female red deer foraging near scent, sniffing 🡪 no response |
|  |  | 7 | Female red deer plus calf foraging 🡪 no response |
| Veluwezoom NP, Netherlands – Wolves absent | Water | 8 | Female red deer plus calf passing by 🡪 no response |
|  |  | 9 | Female red deer plus calf is sniffing and vigilant 🡪 sudden rush  Male red deer is foraging and vigilant 🡪 no response |
|  | All-purpose soap | 10 | Female red deer plus calf walking towards the scent 🡪 sudden rush |
|  |  | 11 | Female red deer passing by 🡪 no response |
|  |  | 12 | Female red deer sniffing, stepping backwards from scent and walking away |
|  |  | 13 | Two female red deer foraging 🡪 no response |
|  | Wolf urine | 14 | Female red deer plus calf foraging. Gets vigilant, start sniffing scent, stepping backwards and walking away |
|  |  | 15 | Male red deer foraging, cleaning body, continues walking 🡪 no response |
|  |  | 16 | Female red deer walking towards scent 🡪 sudden rush |
|  |  | 17 | Female red deer plus calf passing by 🡪 no response |
